# Association between coronary microvascular dysfunction and exercise capacity in dilated cardiomyopathy

**DOI:** 10.1016/j.jocmr.2024.101108

**Published:** 2024-10-18

**Authors:** Abhishek Dattani, Benjamin A. Marrow, Gaurav S. Gulsin, Jian L. Yeo, Amitha Puranik, Emer M. Brady, David Adlam, Anvesha Singh, Mohammedimran M. Ansari, Jayanth R. Arnold, Hui Xue, Peter Kellman, James S. Ware, Gerry P. McCann

**Affiliations:** a Department of Cardiovascular Sciences, University of Leicester and the National Institute for Health and Care Research Leicester Biomedical Research Centre, Leicester, UK; b University Hospitals of Leicester NHS Trust, Leicester, UK; c National Heart, Lung, and Blood Institute, National Institutes of Health, Bethesda, Maryland, USA; d National Heart and Lung Institute & MRC Laboratory of Medical Sciences, Imperial College London, London, UK

**Keywords:** Dilated cardiomyopathy, Myocardial perfusion reserve, Cardiovascular magnetic resonance, Exercise capacity

## Abstract

**Background:**

Aerobic exercise capacity is an independent predictor of mortality in dilated cardiomyopathy (DCM), but the central mechanisms contributing to exercise intolerance in DCM are unknown. The aim of this study was to characterize coronary microvascular function in DCM and determine if cardiovascular magnetic resonance (CMR) measures are associated with aerobic exercise capacity.

**Methods:**

Prospective case-control comparison of adults with DCM and matched controls. Adenosine-stress perfusion CMR to assess cardiac structure, function and automated inline myocardial blood flow quantification, and cardiopulmonary exercise testing to determine peak VO_2_ was performed. Pre-specified multivariable linear regression, including key clinical and cardiac variables, was undertaken to identify independent associations with peak VO_2_.

**Results:**

Sixty-six patients with DCM (mean age 61 years, 47 male) were propensity-matched to 66 controls (mean age 59 years, 47 male) based on age, sex, body mass index, and diabetes. DCM patients had markedly lower peak VO_2_ (19.8 ± 5.5 versus 25.2 ± 7.3 mL/kg/min; P < 0.001). The DCM group had greater left ventricular (LV) volumes, lower systolic function, and more fibrosis compared to controls. In the DCM group, there was similar rest but lower stress myocardial blood flow (1.53 ± 0.49 versus 2.01 ± 0.60 mL/g/min; P < 0.001) and lower myocardial perfusion reserve (MPR) (2.69 ± 0.84 versus 3.15 ± 0.84; P = 0.002). Multivariable linear regression demonstrated that LV ejection fraction, extracellular volume fraction, and MPR, were independently associated with percentage-predicted peak VO_2_ in DCM (R^2^ = 0.531, P < 0.001).

**Conclusion:**

In comparison to controls, DCM patients have lower stress myocardial blood flow and MPR. In DCM, MPR, LV ejection fraction, and fibrosis are independently associated with aerobic exercise capacity.

## Graphical abstract

**Figure:**
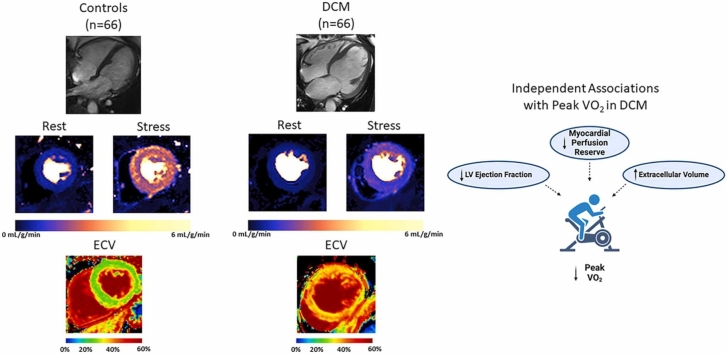
Association between coronary microvascular dysfunction and exercise capacity in dilated cardiomyopathy. In this prospective study using cardiovascular magnetic resonance and cardiopulmonary exercise testing, patients with dilated cardiomyopathy had similar rest but lower stress myocardial blood flow and lower myocardial perfusion reserve compared to controls. In multivariable linear regression incorporating key clinical and imaging parameters, left ventricular ejection fraction, extracellular volume fraction, and myocardial perfusion reserve were independently associated with peak VO_2_ in dilated cardiomyopathy. *DCM* dilated cardiomyopathy, *ECV* extracellular volume fraction

## 1 Introduction

Dilated cardiomyopathy (DCM) is the leading cause of cardiac transplantation and continues to have a high mortality rate [1], [2]. Risk stratification of patients with DCM is difficult, with multiple prediction models of sudden cardiac death failing to reach guidelines [3]. Functional status is often impaired and patients experience frequent heart failure (HF) hospitalizations [4].

Cardiopulmonary exercise testing (CPET) is the gold-standard method for quantification of aerobic exercise capacity (peak oxygen consumption; peak VO_2_), which is an independent predictor of mortality in HF patients [5]. Conventional resting imaging biomarkers have been poor at predicting exercise capacity among those with HF [6].

Cardiovascular magnetic resonance (CMR) is the gold-standard technique for quantification of cardiac volumes and mass, while also allowing assessment of focal and diffuse fibrosis [7], [8]. Recent advances in CMR have allowed the assessment of coronary microvascular dysfunction using automated inline quantification of myocardial blood flow (MBF) [9]. Although DCM is considered to be a predominantly non-ischemic disease, both non-invasive [10] and invasive studies [11] indicate the presence of co-existing coronary microvascular dysfunction. Myocardial perfusion reserve (MPR) is an important predictor of exercise capacity in other cardiac diseases such as aortic stenosis [12], but there are limited data on the prognostic and functional significance of MPR in DCM.

In this study, we aimed to (1) characterize coronary microvascular function in DCM and (2) determine if CMR measures of structure, function, and microvascular function are associated with aerobic exercise capacity. Our hypothesis was that MPR would be an independent predictor of exercise capacity in patients with DCM.

## 2 Methods

### 2.1 Study design and participants

This was a prospective single-center, case-control sub-study of the multi-center GO-DCM study (Defining the Genetics, Biomarkers and Outcomes for Dilated Cardiomyopathy; NCT03843255). Adults with a confirmed diagnosis of DCM based on established age- and sex-specific CMR criteria [13] were recruited from a tertiary referral center in Leicester, United Kingdom. Exclusion criteria were a history of obstructive coronary artery disease (>50% narrowing of any major epicardial coronary artery on invasive or computed tomography coronary angiography), previous percutaneous coronary intervention or coronary bypass surgery, primary valvular heart disease, uncontrolled hypertension, and CMR suggestive of previous myocardial infarction or regional perfusion defects. DCM attributed to chemotherapeutic agents, recreational drug use, excessive alcohol consumption, or inflammatory cardiomyopathies (sarcoid, systemic lupus erythematosus, acute myocarditis) was also excluded. An asymptomatic control group with no history or signs of cardiac disease was selected from concurrently running studies (NCT03132129, ISRCTN42661582) using propensity matching based on age, sex, body mass index, and diabetes status, and underwent the same investigations as the DCM group. Participant’s regular medications were not withheld before any tests as part of this study. Ethical approval was provided by the UK Health Research Authority Research Ethics Committee (18/LO/0692, 17/WM/0192, 14/EM/0056) and all participants provided written informed consent.

### 2.2 General assessments

All participants had demographics, medical history, anthropometric measurements, 12-lead electrocardiogram, and blood samples (including hematocrit) collected. Participants with DCM underwent genotyping to identify pathological variants (Supplemental Table 1).

### 2.3 Cardiopulmonary exercise testing

An incremental symptom-limited CPET (CASE Exercise Testing System, GE HealthCare, Chicago, USA or Vyntus CPX Cart, Vyaire Medical, Illinois, USA) with a bicycle ergometer (eBike Comfort, GE HealthCare, Chicago, USA or VIAsprint 200P, Vyaire Medical, Illinois, USA) was used to assess aerobic exercise capacity. Calibration was performed before each assessment. A 1-minute ramp protocol was used with workload increments calculated based on participant age, sex, weight, and height [14]. Gas analysis was post-processed using appropriate software (Ganshorn LF8, Ganshorn Schiller, Niederlauer, Germany or SentrySuite, Vyaire Medical, Illinois, USA with Cardiosoft ECG software, GE HealthCare, Chicago, USA) using a 30-second rolling mean of breath-by-breath data. Peak VO_2_ was determined as the highest value and percentage-predicted peak VO_2_ was calculated using the Wasserman/Hansen equation [14].

### 2.4 Cardiovascular magnetic resonance

CMR was performed using a 3T scanner (Siemens Skyra/Vida, Erlangen, Germany). Long- and short-axis cine images were acquired using balanced steady-state free precession technique to cover the whole heart. Perfusion imaging was performed at rest and pharmacological stress using 140–210 µg/kg/min of adenosine infused for at least 3 minutes and until symptoms appeared. Rest and stress images were obtained at three short-axis levels (base, mid-ventricular, and apex) following a gadolinium-based contrast agent (0.05 mmol/kg Gadoteric acid, Dotarem, Guerbet, France) using a dual-sequence gradient echo method with inline automated reconstruction and post-processing for MBF quantification [9]. Following rest perfusion imaging, a bolus of 0.1 mmol/kg of contrast was given immediately. Pre- and post-contrast T1 maps were obtained at basal and mid-ventricular level followed by late gadolinium enhancement (LGE) imaging using a segmented approach 15 minutes following the bolus of contrast.

### 2.5 Image analysis

All CMR images were batch analyzed by a single observer (A.D.) using cvi42 (Version 5.10.1, Circle Cardiovascular Imaging, Calgary, Canada) as previously described [15], [16], [17], [18], [19], [20]. The short-axis stack was used to quantify ventricular volumes and mass using the built-in automated contouring tool with adjustments only made for obvious errors [15]. Biplane left atrial volumes were calculated using the four- and two-chamber cine images.

Tissue tracking was used to assess myocardial strain as previously described [20] to calculate global longitudinal and circumferential strain as well as longitudinal and circumferential peak early diastolic strain rate. Blood and myocardial contours were drawn on pre- and post-contrast T1 maps to obtain T1 values for the calculation of extracellular volume fraction (ECV), for which the hematocrit sampled on the same day as the CMR scan was used [18].

All perfusion images were first assessed qualitatively for regional perfusion defects by two experienced observers with their presence leading to exclusion from further analysis. Quantitative perfusion maps were assessed for quality and artifact. Those with poor-quality contouring were manually re-contoured before further analysis. Rest MBF was corrected for resting rate pressure product using the equation: rest MBF/rate pressure product × 10,000. MPR was calculated as the ratio of stress to rest MBF.

LGE images were first qualitatively assessed by two experienced observers for replacement fibrosis which was categorized as present or absent and then further described as infarction or non-infarction. Those patients with infarction seen on LGE were excluded from further analysis. Right ventricular insertion point fibrosis was not deemed pathological and was excluded from the statistical analysis. Quantitative LGE analysis was undertaken using the five standard deviation technique in those with LGE present [19]. The endocardium and epicardium were manually contoured with a further region of interest in normal myocardium remote from the area of enhancement. The total area of enhancement was automatically calculated as the regions with signal intensity greater than five standard deviations above the mean of the region of interest.

### 2.6 Statistical analysis

All continuous variables were assessed for normality. Baseline characteristics were compared using independent T-test, Mann-Whitney U test or Chi-squared test as appropriate. Between group comparisons of CMR and CPET variables were adjusted for key clinical characteristics, which were not already incorporated during propensity matching (CMR: ethnicity and systolic blood pressure; CPET: ethnicity, systolic blood pressure, smoking history, and lung disease). Comparison of perfusion between segments with and without LGE was performed using a linear mixed-effects model accounting for individual patient segmental perfusion.

Correlation of imaging variables with percentage-predicted peak VO_2_ were first assessed using Pearson, Spearman’s rank, or point-biserial correlation in the DCM group to assess for univariate associations and also assessed for collinearity. Multivariable linear regression was used to determine if MPR was independently associated with percentage-predicted peak VO_2_ in the DCM group. This was performed using pre-determined step-wise addition of CMR variables to generate several models: clinical (model 1), structural (model 2), functional (model 3), fibrosis (model 4), and finally the perfusion model (model 5). Model 1 was pre-specified to include New York Heart Association (NYHA) class and N-terminal pro-hormone brain natriuretic peptide (NTproBNP) level due to their recognized functional and prognostic significance. Age, sex, height, and weight were not entered into the models as the use of percentage-predicted peak VO_2_ already accounts for these factors. For models 2–5, selection of variables to be added was based on a combination of the strength of correlation on univariate analyses and the evidence for clinical significance. Statistical analysis was performed using SPSS Statistics (version 28.0, IBM Corp., New York, New York, USA) and power calculations were performed using G*Power (version 3.1.9.6, Heinrich Heine University Düsseldorf, Düsseldorf, Germany) [21]. The linear mixed-effects model was performed using R Statistical Software (version 4.3.1, R Core Team, Vienna, Austria). A P value <0.05 was considered statistically significant throughout.

## 3 Results

### 3.1 Baseline characteristics

**Fig. 1:**
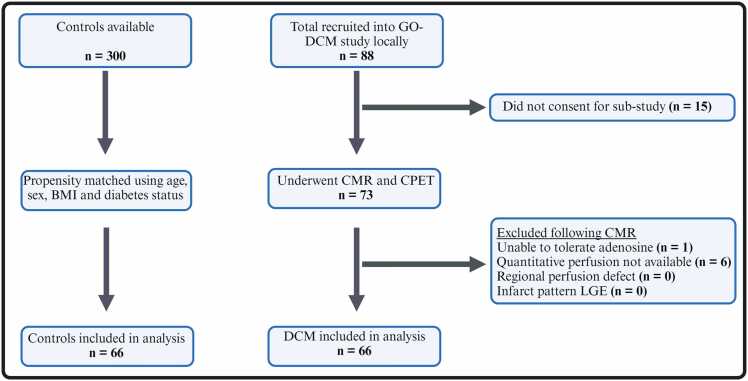
Study flow diagram. Summary of study enrollment and exclusions. *BMI* body mass index, *CMR* cardiovascular magnetic resonance, *CPET* cardiopulmonary exercise testing, *DCM* dilated cardiomyopathy

Following exclusions (Fig. 1), 66 patients with DCM and 66 matched controls were included in this analysis. Baseline characteristics of the groups are shown in Table 1. The groups were well matched for age, sex, body mass index, and diabetes status, but a greater proportion of the DCM group were of White ethnicity compared to controls. The DCM patients had lower blood pressure and resting rate pressure product in comparison to the control group. There was a greater proportion of the DCM group who had asthma, atrial fibrillation/flutter, and hypertension. HF foundational therapies were appropriately prescribed with 97% (64/66) taking renin-angiotensin or combined neprilysin inhibitors, 89% (59/66) on beta-blockers, 65% (43/66) on mineralocorticoid antagonists, and 21% (14/66) on sodium-glucose cotransporter-2 inhibitors. The majority of patients (47/66; 71%) had NYHA class I symptom status. Among the DCM patients, 53 participants had genotyping performed. Of these, 14 (26%) were found to have a genetic variant.

**Table 1:** Baseline characteristics of the DCM and control groups.

	DCM (n = 66)	Control (n = 66)
Age (years)	61.0 ± 10.2	59.0 ± 9.1
Male sex, n (%)	47 (71)	47 (71)
White ethnicity, n (%)	60 (91)*	51 (77)
Height (m)	1.73 ± 0.09	1.72 ± 0.09
Weight (kg)	86.6 ± 17.8	81.6 ± 16.4
Body mass index (kg/m^2^)	28.9 ± 4.8	27.4 ± 4.5
Systolic blood pressure (mmHg)	123 ± 20*	137 ± 19
Diastolic blood pressure (mmHg)	79 ± 10*	85 ± 10
Heart rate (bpm)	64 ± 11	65 ± 9
Rest rate pressure product (mmHg/min)	7591 ± 1677*	8803 ± 1724
*Medical history*		
Hypertension, n (%)	28 (42)*	9 (14)
Hyperlipidemia, n (%)	10 (15)	14 (21)
Diabetes, n (%)	6 (9)	6 (9)
Asthma, n (%)	11 (17)*	2 (3)
COPD, n (%)	1 (1)	0 (0)
Atrial fibrillation/flutter, n (%)	21 (32)*	0 (0)
Smoking, n (%)		
Never	32 (49)	41 (62)
Ex-smoker	30 (46)	22 (33)
Current	4 (6)	3 (5)
NYHA class, n (%)		
1	47 (71)*	66 (100)
2	17 (26)*	0 (0)
3	2 (3)*	0 (0)
Heart failure hospitalization, n (%)	24 (36)*	0 (0)
LBBB, n (%)	21 (32)*	0 (0)
QRS duration (ms)	107 [98-148]*	92 [84-100]
*Drug history*		
ACEi/ ARB, n (%)	37 (56)*	5 (8)
Angiotensin receptor-neprilysin inhibitor, n (%)	27 (41)*	0 (0)
Beta blocker, n (%)	59 (89)*	2 (3)
Mineralocorticoid antagonist, n (%)	43 (65)*	0 (0)
Calcium channel blocker, n (%)	5 (8)	4 (6)
Diuretic, n (%)	26 (39)*	1 (2)
SGLT2i, n (%)	14 (21)*	1 (2)
Statin, n (%)	15 (23)	12 (18)
Antiplatelet, n (%)	6 (9)	1 (2)
Anticoagulation, n (%)	24 (36)*	0 (0)
Biochemistry		
Hemoglobin (g/L)	141 ± 17*	147 ± 12
Hematocrit (L/L)	0.416 [0.386-0.437]	0.425 [0.403-0.443]
eGFR (mL/min/1.73 m^2^)	77.8 ± 15.3	82.4 ± 10.3
NTproBNP (pg/mL)†	1586 [187-2693]*	66 [37-164]

*ACEi* angiotensin converting enzyme inhibitor, *COPD* chronic obstructive pulmonary disease, *DCM* dilated cardiomyopathy, *LBBB* left bundle branch block, *NYHA* New York Heart Association, *SGLT2i* sodium/glucose cotransporter-2 inhibitor

Values presented as mean ± standard deviation, median [interquartile range], or number (%) as appropriate

* P < 0.05 compared to control group

† Available in 47 DCM participants

### 3.2 Imaging data

Table 2 summarizes the CMR data for the cases and controls. The DCM group had higher indexed left ventricular (LV) volumes accompanied by higher LV mass but lower mass-to-volume ratio compared to the controls. LV systolic function (ejection fraction and systolic strain) was lower in the DCM group as was diastolic function (peak early diastolic strain rate). Fourteen (21%) of the DCM patients had recovered LV ejection fraction (>50%), with 37 (56%) of patients still meeting criteria for LV dilatation.

**Table 2:** CMR parameters in the DCM and control groups.

	DCM (n = 66)	Control (n = 66)	Adjusted P value
LV EDVi (mL/m^2^)	116 ± 33	77 ± 13	**<0.001**
LV ESVi (mL/m^2^)	71 ± 35	28 ± 9	**<0.001**
LV SVi (mL/m^2^)	45 ± 11	49 ± 7	0.111
LV CI (L/m^2^)	2.73 ± 0.53	3.26 ± 0.63	**<0.001**
LV EF (%)	41 ± 12	65 ± 7	**<0.001**
LVMi (g/m^2^)	74 ± 14	61 ± 11	**<0.001**
LV M/V (mL/g)	0.65 ± 0.11	0.80 ± 0.12	**<0.001**
Global longitudinal strain (%)	−10.9 ± 3.7	−16.7 ± 2.2	**<0.001**
Global circumferential strain (%)	−11.6 ± 3.8	−18.8 ± 2.5	**<0.001**
Longitudinal PEDSR (s^−1^)	0.45 ± 0.19	0.66 ± 0.17	**<0.001**
Circumferential PEDSR (s^−1^)	0.48 ± 0.18	0.96 ± 0.23	**<0.001**
*Late gadolinium enhancement and ECV*			
LGE present, n (%) LGE percentage of LV mass (%)*	39 (59) 3.8 (2.2-6.0)	2 (3) 3.7 (3.2-4.3)	**<0.001** 0.896
Extracellular volume (%)	29.2 ± 3.6	25.4 ± 1.8	**<0.001**
*Perfusion*			
Rest global MBF (mL/min/g)†	0.79 ± 0.21	0.75 ± 0.21	0.888
Rest endocardial MBF (mL/min/g)†	0.85 ± 0.24	0.76 ± 0.21	0.195
Rest epicardial MBF (mL/min/g)†	0.76 ± 0.24	0.73 ± 0.21	0.794
Rest endocardial:epicardial MBF ratio†	1.13 ± 0.10	1.05 ± 0.05	**<0.001**
Stress global MBF (mL/min/g)	1.53 ± 0.49	2.01 ± 0.60	**<0.001**
Stress endocardial MBF (mL/min/g)	1.46 ± 0.48	1.90 ± 0.57	**<0.001**
Stress epicardial MBF (mL/min/g)	1.56 ± 0.54	2.03 ± 0.62	**<0.001**
Stress endocardial:epicardial MBF ratio	0.95 ± 0.10	0.94 ± 0.08	0.524
Global MPR	2.69 ± 0.84	3.15 ± 0.84	**0.002**
Endocardial MPR	2.44 ± 0.77	2.96 ± 0.78	**<0.001**
Epicardial MPR	2.94 ± 0.92	3.30 ± 0.89	**0.018**
*LA and RV*			
Maximum LAVi (mL/m^2^)	45 ± 20	34 ± 11	**<0.001**
LA EF	47 ± 17	60 ± 8	**<0.001**
RV EDVi (mL/m^2^)	83 ± 18	90 ± 17	0.139
RV ESVi (mL/m^2^)	42 ± 14	42 ± 12	0.858
RV SVi (mL/m^2^)	41 ± 10	48 ± 8	**0.002**
RV EF (%)	50 ± 10	54 ± 6	**0.040**

*CI* cardiac index, *DCM* dilated cardiomyopathy, *EDVi* indexed end-diastolic volume, *ESVi* indexed end-systolic volume, *EF* ejection fraction, *LA* left atrium, *LAVi* left atrial volume index, *LGE* late gadolinium enhancement, *LV* left ventricle, *LVMi* indexed left ventricular mass, *MBF* myocardial blood flow, *MPR* myocardial perfusion reserve, *M/V* mass-to-volume ratio, *PEDSR* peak early diastolic strain rate, *RV* right ventricle, *SVi* indexed stroke volume

Values presented as mean ± standard deviation or number (percentage) as appropriate. P values adjusted for ethnicity and systolic blood pressure. Bold represents P value < 0.05.

* In those with late gadolinium enhancement

† Rest MBFs were corrected for the rate pressure product using the equation: rest MBF/rate pressure product × 10,000

A greater proportion of patients with DCM had replacement fibrosis (LGE) present, all of which was in a non-ischemic distribution, and there was more diffuse fibrosis (ECV) in comparison to the control group. Two control participants had mild non-ischemic LGE present, both of whom had type 2 diabetes and hypercholesterolemia. Among those with LGE, there was no significant difference in quantified LGE between the two groups. In the DCM group, areas of replacement fibrosis were most apparent in the basal-mid septal, inferior, and inferolateral segments (Supplemental Fig. 1).

**Fig. 2:**
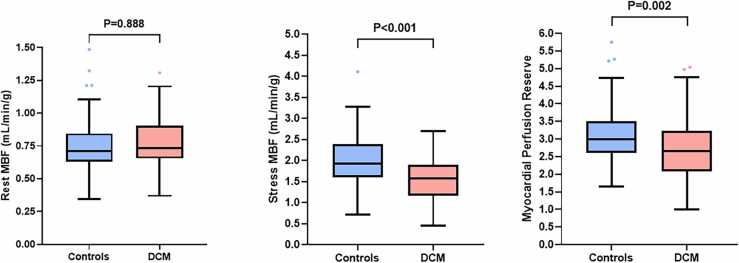
Comparison of quantitative perfusion between the DCM and control groups. Patients with DCM had similar rest (left) but lower stress (middle) MBF and lower myocardial perfusion reserve (right) compared to the control group. P values were adjusted for ethnicity and systolic blood pressure. Boxplots were generated using the Tukey method. *DCM* dilated cardiomyopathy, *MBF* myocardial blood flow

**Fig. 3:**
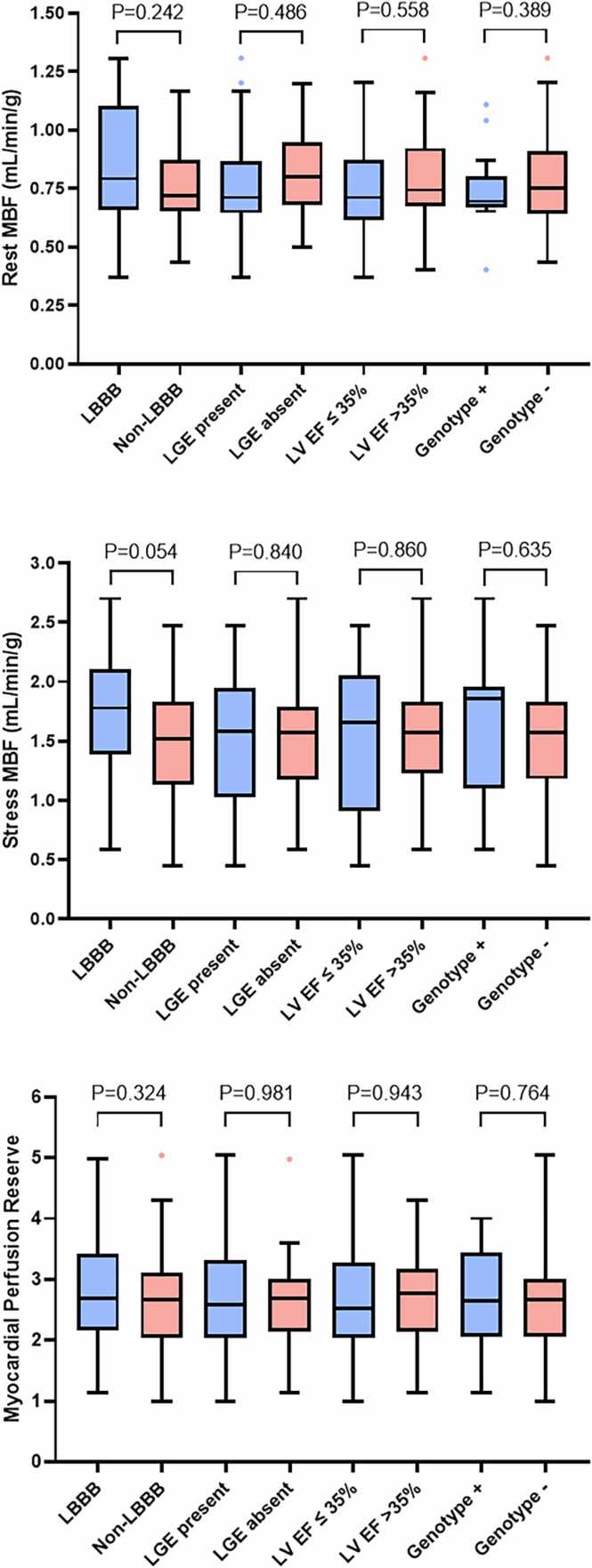
Rest and stress myocardial blood flow and myocardial perfusion reserve in patients with DCM. There was no difference in rest MBF, stress MBF, or MPR in the DCM patients when stratified as LBBB (n = 21) or non-LBBB (n = 45), presence (n = 39) or absence of LGE (n = 27), with an LV EF ≤35% (n = 21) or >35% (n = 45), and genotype positive (n = 14) or negative (n = 39). *EF* ejection fraction, *LBBB* left bundle branch block, *LGE* late gadolinium enhancement, *LV* left ventricular, *MBF* myocardial blood flows, *MPR* myocardial perfusion reserve

There was no significant difference in heart rate (+31% vs +28%; P = 0.243), systolic blood pressure (−3% vs −1%; P = 0.226), or diastolic blood pressure (−2% vs −3%; P = 0.797) response with adenosine infusion in the DCM group compared to the controls. Quantitative perfusion demonstrated similar rest MBF (0.79 ± 0.21 vs 0.75 ± 0.21 mL/min/g; P = 0.888) but lower stress MBF (1.53 ± 0.49 vs 2.01 ± 0.60 mL/g/min; P < 0.001) and MPR (2.69 ± 0.84 vs 3.15 ± 0.84 mL/g/min; P = 0.002) in the DCM group compared to the controls, with consistent patterns at both the endocardial and epicardial levels (Table 2 and Fig. 2). In patients with DCM, MBF and MPR were similar between those with and without left bundle branch block, with and without LGE, those with an ejection fraction below or above 35%, or genotype positive and negative (Fig. 3). Patients with recovered LV ejection fraction had higher rest MBF compared to those without recovered ejection fraction (0.95 ± 0.18 vs 0.74 ± 0.19 mL/min/g; P < 0.001), but there was no difference in stress MBF (1.70 ± 0.42 vs 1.49 ± 0.50 mL/min/g; P = 0.155) or MPR (2.79 ± 0.61 vs 2.67 ± 0.90; P = 0.635). Linear mixed-effects models, however, demonstrated lower rest and stress MBF in segments with LGE compared to segments without LGE in the DCM patients but there was no difference in MPR (Supplemental Table 2).

Sensitivity analysis excluding patients with DCM who had recovered ejection fraction demonstrated consistent findings of similar rest MBF, but lower stress MBF and lower MPR compared to controls (Supplemental Table 3). Furthermore, consistent findings were seen with the exclusion of those DCM patients who had a history of atrial fibrillation/flutter (Supplemental Table 4).

### 3.3 CPET data

The DCM group had lower peak percentage-predicted workload, heart rates, and respiratory exchange ratio although this was >1.0 in both groups (Table 3). Peak VO_2_ (19.8 ± 5.5 vs 25.2 ± 7.3 mL/kg/min; P < 0.001) and percentage-predicted peak VO_2_ (85 ± 19 vs 100 ± 20%; P < 0.001) were lower, and VE/VCO_2_ slope was greater (32.7 ± 7.0 vs 28.0 ± 4.1) in the DCM group compared to controls.

**Table 3:** CPET parameters in the DCM and control groups.

	DCM (n = 66)	Controls (n = 66)	Adjusted P value
Exercise duration (min)	10.0 ± 2.2	10.4 ± 1.6	0.252
Percentage-predicted workload (W)	85 ± 22	109 ± 28	**<0.001**
Peak heart rate (bpm)	135 ± 24	161 ± 26	**<0.001**
Peak VO_2_ (mL/kg/min)	19.8 ± 5.5	25.2 ± 7.3	**<0.001**
Peak RER	1.07 ± 0.08	1.12 ± 0.08	**<0.001**
Percentage-predicted peak VO_2_ (%)	85 ± 19	100 ± 20	**<0.001**
VE/VCO_2_ slope	32.7 ± 7.0	28.0 ± 4.1	**<0.001**

*DCM* dilated cardiomyopathy, *RER* respiratory exchange ratio, *CPET* cardiopulmonary exercise testing

Values presented as mean ± standard deviation

P values adjusted for ethnicity, systolic blood pressure, smoking history, and lung disease

### 3.4 Associations with Peak VO_2_

Univariate correlation analysis in patients with DCM showed significant positive associations between percentage-predicted peak VO_2_ and LV ejection fraction, peak early diastolic strain rate and MPR and inverse associations with LV end-diastolic volume, indexed LV mass, global longitudinal strain, ECV, and presence of LGE (Supplemental Table 5). Neither rest nor stress MBF was associated with percentage-predicted peak VO_2_.

Given significant association between several CMR parameters which are co-linearly related (Supplemental Table 5), forward selection of parameters to be entered into each pre-specified model was based on the strength of the association with peak VO_2_ and prior literature showing significance of each parameter. Multivariable linear regression (Table 4 and Graphical Abstract) demonstrated that LV ejection fraction, ECV, and MPR were independently associated with percentage-predicted peak VO_2_ (R^2^ = 0.531, P < 0.001) in the DCM group. Post-hoc power calculations for the final model showed >99% power (α 0.05, sample size 66, and 6 variables). The same model performed on the control group did not demonstrate significant independent associations of these parameters with percentage-predicted peak VO_2_ (R^2^ = 0.273, P = 0.106). The exploratory addition of a marker of diastolic function (LV peak early diastolic strain rate) did not significantly change the result in the DCM group, although did improve the strength of the final model (R^2^ = 0.660, P < 0.001; Supplemental Table 6). A further exploratory model using the final model to identify associations with weight-adjusted peak VO_2_, rather than percentage-predicted peak VO_2_, which also included age, sex, and weight as covariates demonstrated consistent findings (Supplemental Table 7).

**Table 4:** Multivariable linear regression models for association with percentage-predicted peak VO_2_ in patients with DCM.

	Model 1		Model 2		Model 3		Model 4		Model 5	
	Standardized beta	P value	Standardized beta	P value	Standardized beta	P value	Standardized beta	P value	Standardized beta	P value
NYHA class	**−0.427**	**0.002**	**−0.395**	**0.005**	**−0.371**	**0.006**	**−0.343**	**0.017**	−0.241	0.061
NTproBNP	−0.215	0.109	−0.195	0.148	−0.076	0.580	−0.056	0.694	0.005	0.967
LVMi	**-**	**-**	−0.151	0.271	0.066	0.685	0.115	0.519	0.139	0.382
LV EF	**-**	**-**	**-**	**-**	**0.390**	**0.029**	**0.378**	**0.048**	**0.407**	**0.018**
ECV	**-**	**-**	**-**	**-**	**-**	**-**	−0.223	0.130	**−0.303**	**0.025**
Global MPR	**-**	**-**	**-**	**-**	**-**	**-**	**-**	**-**	**0.404**	**0.002**
R square	0.241		0.263		0.343		0.385		0.531	
Adjusted R square	0.207		0.211		0.280		0.300		0.450	

*ECV* extracellular volume fraction, *LV EF* left ventricular ejection fraction, *LVMi* left ventricular mass index, *NYHA* New York Heart Association, *MPR* myocardial perfusion reserve, *NTproBNP* N-terminal pro brain natriuretic peptide

Sensitivity analysis in the DCM group excluding those with recovered LV ejection fraction demonstrated consistent findings with MPR remaining independently associated with percentage-predicted peak VO2, although LV ejection fraction and ECV were not (Supplemental Table 8). Consistent findings were also seen with the exclusion of patients with atrial fibrillation/flutter (Supplemental Table 9).

## 4 Discussion

In this prospective case-control study, we demonstrate lower stress MBF and MPR in a contemporary group of optimally treated DCM patients in comparison to matched controls. Further, for the first time in DCM, we revealed that MPR, alongside LV ejection fraction and ECV, is independently associated with aerobic exercise capacity, a powerful prognostic marker.

The majority of our DCM patients were on multiple HF remedies with 71% (47/66) in NYHA class I and 21% (14/66) having recovered LV ejection fraction. Nevertheless, there was a high prevalence of replacement fibrosis (59%; 39/66) with elevated mean ECV (29.2%) consistent with significant diffuse fibrosis. This is in contrast to large retrospective cohorts of DCM patients [22], [23]. For example, in one of these studies (n = 659), the participants were functionally worse (only 8% in NYHA class I), had greater LV volumes (indexed end-diastolic volume: 158.5 ± 57.3 mL/m^2^) and poorer LV ejection fraction (28.5 ± 9.5%) with similar proportion of patients with LGE (54%) but less diffuse fibrosis (ECV: 27.7 ± 3.0%) compared to our cohort. These differences in volumes and function may partly represent a selection bias given these studies were retrospectively performed using patients who had been referred for a CMR and therefore participants were less likely to be on traditional HF medications and unlikely to have had significant reverse remodeling. Our study may therefore be more applicable to the general DCM population even following the initiation of medical therapy.

### 4.1 Myocardial perfusion in DCM

Even in early disease, DCM patients are known to have changes in myocardial perfusion with multifactorial processes hypothesized, including both structural changes (capillary rarefaction) and endothelial dysfunction [24]. Although DCM is defined in the absence of epicardial coronary artery disease, microvascular ischemia in the form of chronic hypoperfusion (rest perfusion) and repetitive ischemic insults (stress perfusion) may be a key driver of the disease process and may promote adverse remodeling [25].

Our data of similar rest MBF but reduced stress MBF would support the theory of repetitive ischemic insults but are somewhat contrary to a CMR study which demonstrated higher rest MBF in patients with DCM compared to controls [26]. There are important differences between the two studies which may help explain the discordant results. First, their cohort of DCM patients was younger (age 50 [44–62] years) and had a more severe phenotype (LV ejection fraction 35 [26–46]%, indexed end-diastolic volume 129 [112–171] mL/m^2^) compared to our cohort, which together with the knowledge of the heterogeneity often seen in DCM, may be important. The authors hypothesized that the observed higher rest MBF may be a compensating mechanism for the increased myocardial oxygen demand in DCM, which may explain some of the differences in our results, although there was no association between rest MBF and LV ejection fraction. Surprisingly, their DCM cohort had a lower proportion of patients with LGE (25%) compared to ours. Both our studies have shown that segments with LGE had lower rest MBF which may partly explain the differences in resting MBF. Furthermore, the methodology was substantially different. For example, we used a different field strength (3T scanner) and most importantly, a different technique of MBF quantification meaning that absolute values are not directly comparable. Our study used a gradient echo sequence with higher temporal and better spatial resolution reducing the rate of partial volume effects. The sequence is a dual-echo to account for T2* losses, which allows a more accurate estimation of the arterial input function that is more linearly related to the concentration of contrast [9]. Notably, our technique has shown good agreement with positron emission tomography (PET) [27] and invasive techniques [28]. Our finding of similar rest but reduced stress MBF is consistent with studies that have utilized PET [10], [29], [30] and invasive microvascular assessment [31] in patients with DCM.

At individual patient level, our data showed that segments with LGE had lower rest and stress MBF compared to segments without LGE, which confirms the findings from Gulati et al [26]. This challenges data from studies using PET which have not shown a clear association between MBF and fibrosis in DCM [30], [32] but these studies were relatively small (n = 4 and n = 16). Reduced perfusion within LGE segments could be explained by fibrotic scars leading to excess microvascular remodeling as described in hypertrophic cardiomyopathy [33] or areas of scar having reduced perfusion demand. Importantly, we found no difference in MPR between segments with LGE compared to segments without LGE, and there was no association between MPR and ECV. While this finding is surprising, it is consistent with data in HF with preserved ejection fraction [34] and further challenges the causative role of fibrosis in microvascular dysfunction in HF.

### 4.2 Determinants of exercise capacity

Traditionally, resting measures of systolic cardiac function including LV ejection fraction have been poor predictors of exercise capacity in HF [35], [36]. CMR is the gold-standard technique for volume and function assessment and its multiparametric capabilities simultaneously allow the quantitative assessment of both fibrosis and MPR. With the concurrent use of CPET to assess aerobic exercise capacity in this specific DCM population, we have demonstrated LV ejection fraction being independently associated with peak VO_2_. Much of the previous work has been performed in mixed cohorts of patients with HF of all etiologies which may explain the disparity with our results. Markers of diastolic dysfunction have been associated with exercise capacity [35] and in our data diastolic function (LV peak early diastolic strain rate) showed an association with exercise capacity, although this was not independent of other parameters.

#### 4.2.1 Myocardial perfusion reserve

This is the largest study, and the first using CMR, to assess the relationship between MPR and exercise capacity in patients with DCM. We showed that MPR was independently associated with aerobic exercise capacity, and together in a model containing clinical, structural, functional, and fibrosis parameters, explains 53% of the variation in exercise capacity. To our knowledge, there have been only two small (n = 22 and n = 20) non-CMR studies assessing the role of MPR in exercise capacity in this patient population [10], [37]. One of these studies used a different methodology to calculate oxygen consumption (carbon-11 acetate clearance rate during submaximal dobutamine infusion) and although they found an association between MPR and oxygen consumption, they did not perform multivariable linear regression to determine independence of the association. The second study did not perform adjustment for age and sex which are strong predictors of exercise capacity. Both of these studies were performed using PET which has important limitations, such as the lower spatial resolution compared to CMR. This becomes particularly relevant given the thin myocardium often seen in DCM and therefore a study using CMR was imperative.

Independent association between MPR and aerobic exercise capacity has been shown in other conditions, such as aortic stenosis [12] and diabetic cardiomyopathy [16] suggesting that MPR is an important predictor of exercise capacity across a spectrum of cardiovascular diseases. Exercise increases cardiac workload as determined by the rate pressure product leading to increased myocardial oxygen demand. Myocardial oxygen consumption is a product of coronary blood flow and the arterial-venous oxygen difference. Our data and other studies have shown impaired stress MBF and MPR in DCM suggesting an inability to increase coronary blood flow at point of need, as evidenced by a striking 24% reduction in mean stress MBF in our DCM patients compared to controls. This could result in impaired cardiac work during exercise and the inability to increase stroke volume with a resultant decrease in exercise capacity.

We propose that given there was no association between MPR and LV ejection fraction in our cohort, MPR may serve additional prognostic information beyond systolic function in some cohorts of DCM, which is supported by its independent association with peak VO_2_ seen in our study. MPR may act as a potential therapeutic target and strategies to improve endothelial dysfunction should be investigated as a means to alleviate exercise intolerance in DCM.

#### 4.2.2 Extracellular volume fraction

ECV measures the volume of cardiac tissue not taken up by cardiomyocytes and is a surrogate for diffuse interstitial fibrosis, which has been validated histologically in DCM [38]. To our knowledge, no studies have assessed the relationship between ECV and exercise capacity in DCM. In a cohort of hypertrophic cardiomyopathy patients, however, the presence of LGE on CMR, which assesses replacement fibrosis, was found to be independently associated with poorer exercise capacity [39]. While their study did not assess ECV, in our DCM cohort we also demonstrated the association between the presence of LGE and aerobic exercise capacity (r = −0.265), and the presence of LGE was associated with ECV (r = 0.430). LGE has been linked to outcomes in DCM [40], and ECV [41], [42] has prognostic implications in various cardiovascular diseases including recently in DCM [43].

## 5 Limitations

This prospective study is the first to assess the association between multiparametric CMR variables and exercise capacity in a contemporary group of well-treated DCM patients and matched controls. This analysis had a pre-specified hypothesis to determine the role of myocardial perfusion in aerobic exercise capacity and the categories of the regression models used were also pre-specified with data-driven selection of individual parameters. Strict exclusion criteria were applied to truly represent the DCM population as defined by international guidelines. The use of propensity-matched controls and statistical adjustment avoided significant confounder bias during case-control comparison. A significant minority of our DCM patients had recovered ejection fraction, adding to the generalizability and potential impact of these data on the wider population of treated DCM patients.

The modest size of the DCM cohort limited the number of variables insertable into the model without overfitting, despite this our study is currently the largest cohort of patients with DCM studied with an aim to assess the association between MPR and exercise capacity. Although no differences were seen in MBFs based on genotyping, this was limited by sample size and the variety of causative genes identified. This study is a cross-sectional analysis of cases and controls and although patients have consented to long-term follow-up, meaningful outcome data will not be available for several more years.

## 6 Conclusions

In this prospective study of patients with DCM, lower stress MBF and MPR were observed in comparison to matched controls. For the first time, we demonstrated that MPR, alongside LV ejection fraction and ECV, was independently associated with aerobic exercise capacity. Longitudinal studies assessing whether strategies to improve MPR result in increased exercise capacity are warranted.

## Funding

A.D. received funding from the 10.13039/501100000274British Heart Foundation through a Clinical Research Training Fellowship (FS/CRTF/20/24069). J.L.Y., E.M.B., and G.P.M. received funding from the National Institute for Health and Care Research (NIHR) through a Research Professorship award (RP-2017-08-ST2-007). The GO-DCM study was funded by the 10.13039/501100000274British Heart Foundation (SP/17/11/32885). D.A. was supported by the British Heart Foundation (BHF) PG/13/96/30608, the NIHR rare disease translational collaboration, and BeatSCAD. J.S.W. received funding from Medical Research Council (UK), Sir Jules Thorn Charitable Trust (21JTA), 10.13039/501100000274British Heart Foundation (RE/18/4/34215), has received research support from 10.13039/100008021Bristol Myers Squibb, and has acted as a consultant for MyoKardia, Pfizer, Foresite Labs, Health Lumen, and Tenaya Therapeutics.

## Author contributions

**Amitha Puranik:** Writing—review and editing, Methodology, Formal analysis. **Jian L. Yeo:** Supervision, Methodology, Investigation. **David Adlam:** Writing—review and editing, Funding acquisition, Conceptualization. **Emer M. Brady:** Writing—review and editing, Formal analysis. **Mohammedimran M. Ansari:** Supervision, Methodology. **Anvesha Singh:** Writing—review and editing. **Hui Xue:** Resources, Methodology, Investigation. **Jayanth R. Arnold:** Writing—review and editing, Conceptualization. **Abhishek Dattani:** Writing—review and editing, Writing—original draft, Supervision, Methodology, Investigation, Formal analysis, Data curation. **Gaurav S. Gulsin:** Writing—review and editing, Methodology, Investigation. **Benjamin A. Marrow:** Writing—review and editing, Supervision, Methodology, Investigation, Funding acquisition, Conceptualization. **James S. Ware:** Writing—review and editing, Funding acquisition, Formal analysis. **Peter Kellman:** Resources, Methodology, Investigation. **Gerry P. McCann:** Writing—review and editing, Supervision, Resources, Methodology, Investigation, Formal analysis, Conceptualization.

## Declaration of competing interests

The authors declare that they have no known competing financial interests or personal relationships that could have appeared to influence the work reported in this paper. The author Gerry P. McCann is an Editorial Board Member for *Journal of Cardiovascular Magnetic Resonance* and was not involved in the editorial review or the decision to publish this article.
