# Supplementary material for: Association between coronary microvascular dysfunction and exercise capacity in dilated cardiomyopathy
Source: J Cardiovasc Magn Reson. 2024 Oct 18;26(2):101108. doi: 10.1016/j.jocmr.2024.101108 (PMC11647499; doi:10.1016/j.jocmr.2024.101108)
Supplement: Supplementary file 1 — Supplementary material [file mmc1.docx]

Association Between Coronary Microvascular Dysfunction and Exercise Capacity in Dilated Cardiomyopathy

**Supplemental Table 1:** Genes sequenced and their corresponding proteins

| Gene | Protein |
| --- | --- |
| BAG3 | BCL2-associated athanogene 3 |
| DES | Desmin |
| FLNC | Filamin C |
| LMNA | Lamin A/C |
| MYH7 | Myosin heavy chain 7 |
| PLN | Phospholamban |
| RBM20 | RNA-binding motif protein 20 |
| SCN5A | Sodium voltage-gated channel, α subunit 5 |
| TNNC1 | Troponin C |
| TNNT2 | Troponin T2 |
| TTN | Titin |
| DSP | Desmoplakin |
| ACTC1 | α Actin |
| ACTN2 | Actinin α2 |
| JPH2 | Junctophilin 2 |
| NEXN | Nexilin F-actin–binding protein |
| TNNI3 | Troponin I |
| TPM1 | Tropomyosin 1 |
| VCL | Vinculin |

**Supplemental Figure 1:** Distribution of Late Gadolinium Enhancement in Patients with Dilated Cardiomyopathy with scar on Late Gadolinium Enhancement (n=39/66)

**
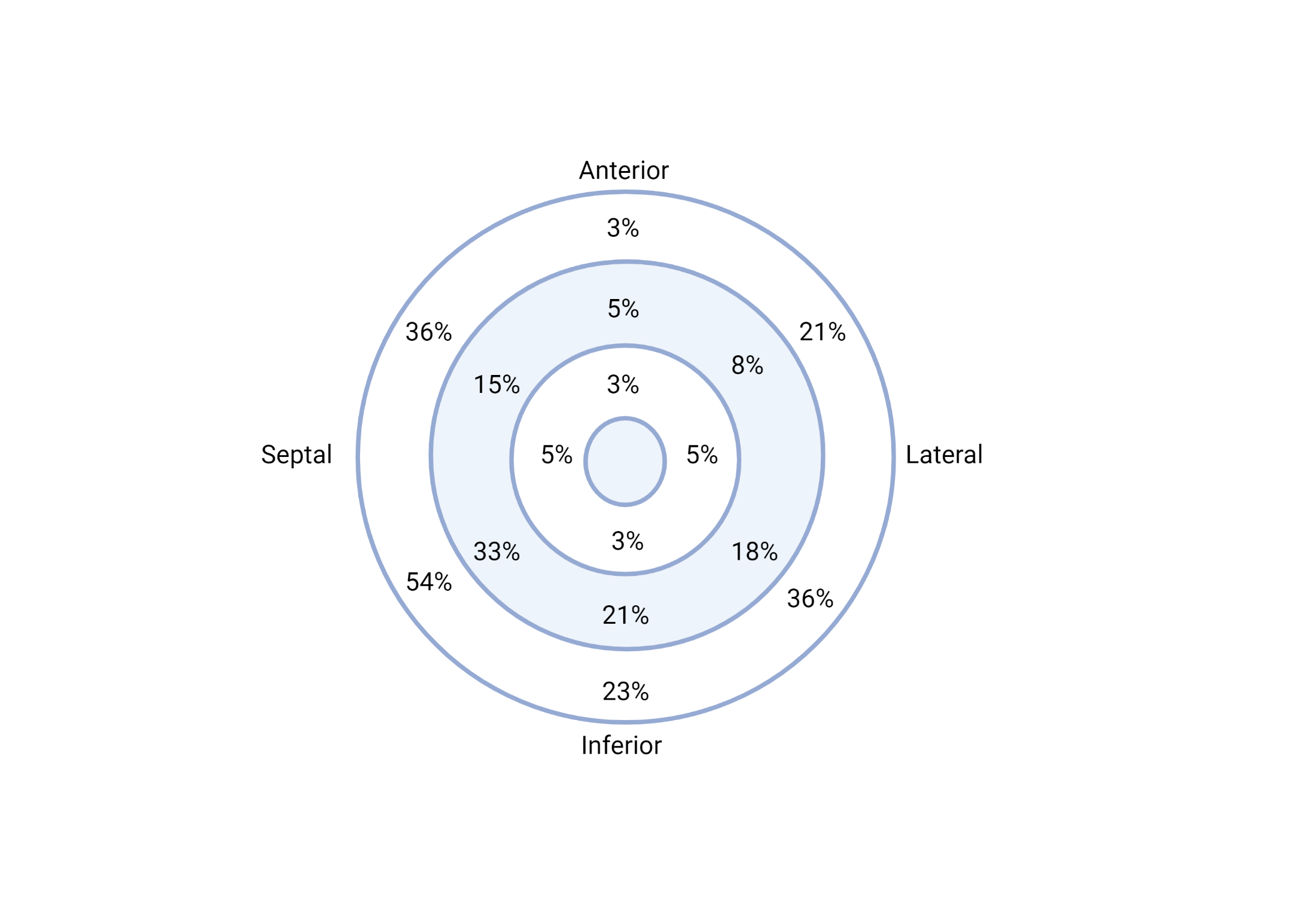
**

| Supplemental Table 2: Myocardial Perfusion in Segments with and without LGE in Patients with Dilated Cardiomyopathy (n=66) | | | |
| --- | --- | --- | --- |
|  | **Segments with LGE**  **(n=112)** | **Segments without LGE**  **(n=944)** | **P value** |
| Rest MBF (mL/min/g) | 0.72 (0.67-0.78) | 0.79 (0.74-0.84) | **<0.001** |
| Stress MBF (mL/min/g) | 1.38 (1.25-1.51) | 1.55 (1.43-1.67) | **<0.001** |
| MPR | 2.71 (2.47-2.96) | 2.76 (2.55-2.97) | 0.496 |

Abbreviations: MBF = myocardial blood flow, MPR = myocardial perfusion reserve, LGE = late gadolinium enhancement.

Values presented as adjusted means (95% confidence interval).

| Supplemental Table 3: Comparison of the groups with exclusion of DCM patients with recovered LV ejection fraction | | | |
| --- | --- | --- | --- |
|  | DCM with EF<50%  (n=52) | Control  (n=66) | P value |
| Age (years) | 62.6 ± 9.2 | 59.3 ± 9.1 | 0.061 |
| Male sex, n (%) | 37 (71) | 47 (71) | 0.994 |
| Body mass index (kg/m^2^) | 28.8 ± 5.1 | 27.4 ± 4.4 | 0.126 |
| CPET |  |  |  |
| Peak VO_2_ (mL/kg/min) | 18.5 ± 4.5 | 25.2 ± 7.3 | **<0.001** |
| CMR |  |  |  |
| LV EDVi (mL/m^2^) | 122 ± 34 | 77 ± 13 | **<0.001** |
| LV EF (%) | 37 ± 10 | 65 ± 7 | **<0.001** |
| Global Rest MBF ^•^ (mL/min/g) | 0.74 ± 0.19 | 0.75 ± 0.21 | 0.226 |
| Global Stress MBF (mL/min/g) | 1.49 ± 0.50 | 2.01 ± 0.60 | **<0.001** |
| Global MPR | 2.67 ± 0.90 | 3.15 ± 0.84 | **0.004** |
| Extracellular volume (%) | 29.9 ± 3.5 | 25.4 ± 1.8 | **<0.001** |

Abbreviations: DCM = dilated cardiomyopathy, EDVi = indexed end-diastolic volume, EF = ejection fraction, LV= left ventricle, MBF = myocardial blood flow, MPR = myocardial perfusion reserve.

Values presented as mean ± standard deviation. P values adjusted for ethnicity and systolic blood pressure

**^•^** Rest MBF was corrected for the rate pressure product using the equation: rest MBF/rate pressure product x 10,000

| Supplemental Table 4: Comparison of the groups with exclusion of DCM patients with history of atrial fibrillation/flutter | | | |
| --- | --- | --- | --- |
|  | DCM  (n=45) | Control  (n=66) | P value |
| Age (years) | 59.5 ± 9.7 | 59.3 ± 9.1 | 0.941 |
| Male sex, n (%) | 29 (64) | 47 (71) | 0.451 |
| Body mass index (kg/m^2^) | 29.0 ± 4.5 | 27.4 ± 4.4 | 0.062 |
| CPET |  |  |  |
| Peak VO_2_ (mL/kg/min) | 20.0 ± 5.7 | 25.2 ± 7.3 | **<0.001** |
| CMR |  |  |  |
| LV EDVi (mL/m^2^) | 119 ± 35 | 77 ± 13 | **<0.001** |
| LV EF (%) | 42 ± 11 | 65 ± 7 | **<0.001** |
| Global Rest MBF ^•^ (mL/min/g) | 0.80 ± 0.19 | 0.75 ± 0.21 | 0.920 |
| Global Stress MBF (mL/min/g) | 1.56 ± 0.47 | 2.01 ± 0.60 | **<0.001** |
| Global MPR | 2.77 ± 0.86 | 3.15 ± 0.84 | **0.013** |
| Extracellular volume (%) | 29.4 ± 3.3 | 25.4 ± 1.8 | **<0.001** |

Abbreviations: DCM = dilated cardiomyopathy, EDVi = indexed end-diastolic volume, EF = ejection fraction, LV= left ventricle, MBF = myocardial blood flow, MPR = myocardial perfusion reserve.

Values presented as mean ± standard deviation. P values adjusted for ethnicity and systolic blood pressure

**^•^** Rest MBF was corrected for the rate pressure product using the equation: rest MBF/rate pressure product x 10,000

| Supplemental Table 5: Correlation Matrix Showing Univariate Associations with Percentage Predicted Peak VO2 in Patients with Dilated Cardiomyopathy | | | | | | | | | | | | | | | | | | | | |
| --- | --- | --- | --- | --- | --- | --- | --- | --- | --- | --- | --- | --- | --- | --- | --- | --- | --- | --- | --- | --- |
|  | **% Peak**  **VO2** | **LVEDVi** | **LVSVi** | **LVEF** | **LVMi** | **LVM/V** | **GLS** | **GCS** | **lPEDSR** | **cPEDSR** | **LAVi** | **LAEF** | **RVEDVi** | **RVSVi** | **RVEF** | **ECV** | **LGE** | **Rest**  **MBF** | **Stress**  **MBF** | **MPR** |
| % Peak VO2 |  | -.302^*^ | .485^**^ | .496^**^ | -.330^**^ | 0.019 | .470^**^ | .476^**^ | .387^**^ | .442^**^ | -0.061 | .299^*^ | 0.087 | .413^**^ | .361^**^ | -.310^*^ | -.265^*^ | 0.030 | 0.203 | .250^*^ |
| LVEDVi | -.302^*^ |  | -0.047 | -.731^**^ | .798^**^ | -.668^**^ | -.568^**^ | -.617^**^ | -.295^*^ | -.401^**^ | 0.167 | -.289^*^ | .401^**^ | 0.078 | -.305^*^ | .333^*^ | .301^*^ | 0.032 | 0.066 | 0.013 |
| LVSVi | .485^**^ | -0.047 |  | .647^**^ | -0.126 | -0.173 | .625^**^ | .608^**^ | .380^**^ | .433^**^ | 0.088 | .369^**^ | .432^**^ | .863^**^ | .561^**^ | -0.141 | -0.166 | 0.215 | 0.134 | 0.057 |
| LVEF | .496^**^ | -.731^**^ | .647^**^ |  | -.660^**^ | .408^**^ | .831^**^ | .862^**^ | .454^**^ | .571^**^ | -0.140 | .482^**^ | -0.046 | .474^**^ | .599^**^ | -.364^**^ | -.351^**^ | 0.233 | 0.026 | 0.004 |
| LVMi | -.330^**^ | .798^**^ | -0.126 | -.660^**^ |  | -0.132 | -.650^**^ | -.593^**^ | -.440^**^ | -.484^**^ | 0.055 | -0.183 | 0.224 | -0.089 | -.317^**^ | .337^**^ | 0.208 | -0.098 | -0.075 | -0.043 |
| LVM/V | 0.019 | -.668^**^ | -0.173 | .408^**^ | -0.132 |  | 0.156 | .294^*^ | -0.101 | 0.005 | -.300^*^ | .259^*^ | -.450^**^ | -.298^*^ | 0.107 | -0.193 | -0.229 | -0.093 | -.251^*^ | -0.117 |
| GLS | .470^**^ | -.568^**^ | .625^**^ | .831^**^ | -.650^**^ | 0.156 |  | .858^**^ | .587^**^ | .499^**^ | -0.121 | .518^**^ | 0.029 | .555^**^ | .624^**^ | -.288^*^ | -.340^**^ | 0.239 | 0.104 | 0.055 |
| GCS | .476^**^ | -.617^**^ | .608^**^ | .862^**^ | -.593^**^ | .294^*^ | .858^**^ |  | .495^**^ | .595^**^ | -0.243 | .616^**^ | 0.012 | .569^**^ | .651^**^ | -.424^**^ | -.395^**^ | .290^*^ | 0.117 | 0.034 |
| lPEDSR | .387^**^ | -.295^*^ | .380^**^ | .454^**^ | -.440^**^ | -0.101 | .587^**^ | .495^**^ |  | .717^**^ | 0.230 | 0.129 | 0.174 | .328^*^ | 0.243 | -0.209 | -0.175 | .289^*^ | 0.123 | -0.151 |
| cPEDSR | .442^**^ | -.401^**^ | .433^**^ | .571^**^ | -.484^**^ | 0.005 | .499^**^ | .595^**^ | .717^**^ |  | 0.162 | 0.165 | 0.241 | .378^**^ | 0.201 | -.535^**^ | -.310^*^ | .313^*^ | 0.089 | -0.161 |
| LAVi | -0.061 | 0.167 | 0.088 | -0.140 | 0.055 | -.300^*^ | -0.121 | -0.243 | 0.230 | 0.162 |  | -.620^**^ | .355^**^ | 0.060 | -.275^*^ | 0.192 | 0.059 | -0.043 | 0.029 | -0.038 |
| LAEF | .299^*^ | -.289^*^ | .369^**^ | .482^**^ | -0.183 | .259^*^ | .518^**^ | .616^**^ | 0.129 | 0.165 | -.620^**^ |  | -0.178 | .348^**^ | .618^**^ | -0.242 | -0.099 | 0.184 | 0.123 | 0.096 |
| RVEDVi | 0.087 | .401^**^ | .432^**^ | -0.046 | 0.224 | -.450^**^ | 0.029 | 0.012 | 0.174 | 0.241 | .355^**^ | -0.178 |  | .577^**^ | -.297^*^ | 0.126 | 0.110 | 0.063 | 0.026 | -0.038 |
| RV_SVi | .413^**^ | 0.078 | .863^**^ | .474^**^ | -0.089 | -.298^*^ | .555^**^ | .569^**^ | .328^*^ | .378^**^ | 0.060 | .348^**^ | .577^**^ |  | .582^**^ | -0.170 | -0.108 | 0.169 | 0.071 | 0.018 |
| RVEF | .361^**^ | -.305^*^ | .561^**^ | .599^**^ | -.317^**^ | 0.107 | .624^**^ | .651^**^ | 0.243 | 0.201 | -.275^*^ | .618^**^ | -.297^*^ | .582^**^ |  | -0.220 | -0.228 | 0.193 | 0.114 | 0.110 |
| ECV | -.310^*^ | .333^*^ | -0.141 | -.364^**^ | .337^**^ | -0.193 | -.288^*^ | -.424^**^ | -0.209 | -.535^**^ | 0.192 | -0.242 | 0.126 | -0.170 | -0.220 |  | .430^**^ | -0.054 | 0.138 | 0.179 |
| LGE | -.265^*^ | .301^*^ | -0.166 | -.351^**^ | 0.208 | -0.229 | -.340^**^ | -.395^**^ | -0.175 | -.310^*^ | 0.059 | -0.099 | 0.110 | -0.108 | -0.228 | .430^**^ |  | -0.087 | -0.025 | -0.003 |
| Rest MBF | 0.030 | 0.032 | 0.215 | 0.233 | -0.098 | -0.093 | 0.239 | .290^*^ | .289^*^ | .313^*^ | -0.043 | 0.184 | 0.063 | 0.169 | 0.193 | -0.054 | -0.087 |  | .360^**^ | -0.115 |
| Stress MBF | 0.203 | 0.066 | 0.134 | 0.026 | -0.075 | -.251^*^ | 0.104 | 0.117 | 0.123 | 0.089 | 0.029 | 0.123 | 0.026 | 0.071 | 0.114 | 0.138 | -0.025 | .360^**^ |  | .715^**^ |
| MPR | .250^*^ | 0.013 | 0.057 | 0.004 | -0.043 | -0.117 | 0.055 | 0.034 | -0.151 | -0.161 | -0.038 | 0.096 | -0.038 | 0.018 | 0.110 | 0.179 | -0.003 | -0.115 | .715^**^ |  |

Abbreviations: LV=left ventricle, EDVi=indexed end-diastolic volume, SVi=indexed stroke volume, EF=ejection fraction, LVMi=LV mass index, M/V=mass-to-volume ratio, GLS=global longitudinal strain (absolute value), GCS=global circumferential strain (absolute value), lPEDSR=longitudinal peak early diastolic strain rate, cPLDSR=circumferential peak early diastolic strain rate, LAVi=left atrial volume index, RV=right ventricle, ECV=extracellular volume fraction, LGE=late gadolinium enhancement, MBF=myocardial blood flow, MPR=myocardial perfusion reserve

| Supplemental Table 6: Multivariable Regression Models for Association with Percentage Predicted Peak VO_2_ in Patients with Dilated Cardiomyopathy with the Addition of a Marker of Diastolic Function. | | | | | | | | | | |
| --- | --- | --- | --- | --- | --- | --- | --- | --- | --- | --- |
|  | **Model 1** | | **Model 2** | | **Model 3** | | **Model 4** | | **Model 5** | |
|  | Standardized  Beta | P value | Standardized  Beta | P value | Standardized  Beta | P value | Standardized  Beta | P value | Standardized  Beta | P value |
| NYHA class | **-0.427** | **0.002** | **-0.395** | **0.005** | **-0.424** | **0.003** | **-0.399** | **0.006** | **-0.292** | **0.022** |
| NTproBNP | -0.215 | 0.109 | -0.195 | 0.148 | -0.016 | 0.909 | 0.010 | 0.945 | 0.073 | 0.554 |
| LVMi | **-** | **-** | -0.151 | 0.271 | 0.072 | 0.691 | 0.160 | 0.422 | 0.273 | 0.126 |
| LV EF | **-** | **-** | **-** | **-** | **0.429** | **0.031** | 0.378 | 0.083 | **0.449** | **0.021** |
| Longitudinal PEDSR | **-** | **-** | **-** | **-** | 0.166 | 0.244 | 0.239 | 0.126 | 0.263 | 0.054 |
| ECV | **-** | **-** | **-** | **-** | **-** | **-** | -0.247 | 0.107 | **-0.355** | **0.012** |
| Global MPR | **-** | **-** | **-** | **-** | **-** | **-** | **-** | **-** | **0.400** | **0.003** |
| R square | 0.241 |  | 0.263 |  | 0.458 |  | 0.527 |  | 0.660 |  |
| Adjusted R square | 0.207 |  | 0.211 |  | 0.378 |  | 0.426 |  | 0.572 |  |

Abbreviations: ECV = extracellular volume fraction, LV EF = left ventricular ejection fraction, LVMi = left ventricular mass index, NYHA = New York Heart Association, PEDSR = peak early diastolic strain rate, MPR = myocardial perfusion reserve.

|  | Standardized Beta | P value |
| --- | --- | --- |
| Age | -0.623 | **<0.001** |
| Male sex | 0.499 | **<0.001** |
| Weight | -0.391 | **0.003** |
| NYHA class | -0.143 | 0.155 |
| NTproBNP | 0.039 | 0.737 |
| LVMi | -0.058 | 0.659 |
| LV EF | 0.239 | 0.078 |
| ECV | -0.238 | **0.030** |
| Global MPR | 0.267 | **0.009** |
| R square | 0.750 |  |
| Adjusted R square | 0.680 |  |

**Supplemental Table 7:** Multivariable regression Model for Association with weight-adjusted Peak VO_2_ in Patients with Dilated Cardiomyopathy

Abbreviations: ECV = extracellular volume fraction, LV EF = left ventricular ejection fraction, LVMi = left ventricular mass index, NYHA = New York Heart Association, MPR = myocardial perfusion reserve

**Supplemental Table 8:** Multivariable Regression Model for Association with Percentage Predicted Peak VO_2_ in Patients with Dilated Cardiomyopathy excluding patients with recovered ejection fraction

|  | Standardized  Beta | P value |
| --- | --- | --- |
| NYHA class | -0.172 | 0.282 |
| NTproBNP | -0.005 | 0.972 |
| LVMi | 0.075 | 0.689 |
| LV EF | 0.193 | 0.309 |
| ECV | -0.313 | 0.055 |
| Global MPR | **0.514** | **0.002** |

Abbreviations: ECV = extracellular volume fraction, EF = ejection fraction, LV = left ventricle, LVMi = left ventricular mass index, NYHA = New York Heart Association, MPR = myocardial perfusion reserve.

**Supplemental Table 9:** Multivariable Regression Model for Association with Percentage Predicted Peak VO2 in Patients with Dilated Cardiomyopathy Excluding Patients with Atrial Fibrillation/Flutter.

|  | Standardized  Beta | P value |
| --- | --- | --- |
| NYHA class | **-0.388** | **0.039** |
| NTproBNP | -0.060 | 0.763 |
| LVMi | 0.202 | 0.343 |
| LV EF | 0.338 | 0.152 |
| ECV | -0.245 | 0.208 |
| Global MPR | **0.371** | **0.041** |

Abbreviations: ECV = extracellular volume fraction, EF = ejection fraction, LV = left ventricle, LVMi = left ventricular mass index, NYHA = New York Heart Association, MPR = myocardial perfusion reserve.
